# Challenges and emerging strategies for genome-wide evaluation of loss of imprinting in cancer

**DOI:** 10.3389/bjbs.2026.16709

**Published:** 2026-06-09

**Authors:** Muhammad Talal Amin, Louis Coussement, Tim De Meyer

**Affiliations:** 1 Department of Data Analysis and Mathematical Modelling, Faculty of Bioscience Engineering, Ghent University, Ghent, Belgium; 2 Cancer Research Institute Ghent (CRIG), Ghent, Belgium; 3 Department of Bioscience and Technology, Khwaja Fareed University of Engineering and Information Technology, Rahim Yar Khan, Pakistan; 4 Bioinformatics Institute Ghent N2N, Ghent University, Ghent, Belgium

**Keywords:** cancer, genomic imprinting, loss of imprinting (LOI), multi-omics, single cell sequencing

## Abstract

Genomic imprinting is the phenomenon in which only a single allele of a gene is expressed based on its parental origin, thereby deviating from the typical biallelic expression of autosomal genes. It is meticulously controlled by epigenetic mechanisms, particularly DNA methylation. Imprinted loci are crucial for regulating growth during early development, and anomalies in imprinting can lead to congenital syndromes such as Beckwith-Wiedemann’s and Prader-Willi’s. Similarly, many cancers exhibit dysregulated imprinting patterns, putatively contributing to tumour growth. Yet, the assessment of imprinting in cancer is complex due to technical challenges, impeding clinical research and the translation of novel insight to the clinic. This review starts with a general introduction to imprinting, its (dys)regulation and key clinical findings in cancer and beyond. Then, we summarize common methods used to characterize normal imprinting and aberrations in cancer. Subsequently, we discuss how the interpretation of such findings is complicated by technical challenges, such as tumour impurity, the requirement for heterozygosity to distinguish between maternal and paternal alleles and the presence of tissue- and transcript-specific imprinting patterns. We further delve into state-of-the-art methods able to mitigate these challenges. Finally, we discuss how future methodological innovations, particularly by integrating single-cell and single-molecule based methods, may further facilitate a straightforward characterization of imprinting dysregulation and its underlying causes, and guide the development of clinical tests. Thus, by integrating recent advances and proposing innovative approaches, our review aims to provide a comprehensive overview for cancer researchers and clinicians to facilitate cancer imprinting research and its translation to the clinic.

## Introduction

Genomic imprinting is a unique epigenetic process that results in the monoallelic expression of a gene based on its parental origin. This contrasts classical Mendelian inheritance, which assumes that both alleles of the gene equally contribute to an organism’s phenotype [1, 2]. The earliest hints of imprinting emerged from studies on nuclear transplantation experiments in mice. Developmental biologists observed that embryos with uniparental genomes, where both sets of chromosomes were inherited from the same parent, failed to develop normally [3]. These findings suggested that certain genes required contributions from both the maternal and paternal genome, contradicting the expectation that two gene copies, independent of parental origin, are sufficient. Further research pinpointed specific genes such as *IGF2* and *H19*, demonstrating parent-of-origin specific expression, leading to the formal recognition of genomic imprinting as a key epigenetic phenomenon [2]. Since then, over 100 imprinted genes have been identified in humans and mice, with many of them playing vital roles in embryonic growth, metabolism, and neurological development [4].

The most widely accepted evolutionary theory explaining the origin of genomic imprinting is the parental conflict hypothesis, even though there are counterexamples and alternative theories [5]. Unlike most other epigenetic regulatory mechanisms that modulate gene expression in a cell-type-specific or environment dependent manner, imprinting is established in germ cells and generally maintained throughout an organism’s lifetime. The parental conflict hypothesis therefore posits that paternally expressed genes promote growth and therefore offspring viability during early development, whereas maternally expressed genes counterbalance growth stimulation to also preserve maternal health [5].

It is therefore not unexpected that imprinting disruption typically leads to aberrant expression of growth affecting genes, causing growth-related syndromes [6], but also promoting cancer growth [7], making it particularly relevant for clinical research. Moreover, clinical test strategies and treatments targeting imprinting loss are emerging [8–12]. Yet only few imprinting-based cancer biomarkers and targets have been translated to the clinic. We argue that this can be particularly attributed to methodological bottlenecks.

Hence, this review aims to discuss well-established and innovative methodologies for the characterization of imprinting and its dysregulation, emphasizing their application as well as limitations for clinical cancer research. Given our focus on the methodological aspects of genomic imprinting research, we only provide a basic overview of normal imprinting and its dysregulation in cancer to introduce the methodological bottlenecks encountered in clinical (cancer) research. For a more comprehensive and detailed overview of the molecular mechanisms controlling imprinting, its deregulation in general and in specific genes/diseases, we refer to the cited literature.

## Epigenetic regulation of imprinting

### DNA methylation as key player of imprinting regulation

Genomic imprinting is regulated by epigenetic modifications that establish and maintain parental allele-specific expression patterns. Imprinted genes are often found clustered within genomic loci regulated by Imprinting Control Regions (ICRs), which are *cis*-regulatory elements that epigenetically govern allele-specific gene expression. At imprinted genes, ICRs and other regulatory elements typically feature about 50% DNA methylation, reflecting parent-of-origin dependent regulation [3].

**FIGURE 1:**
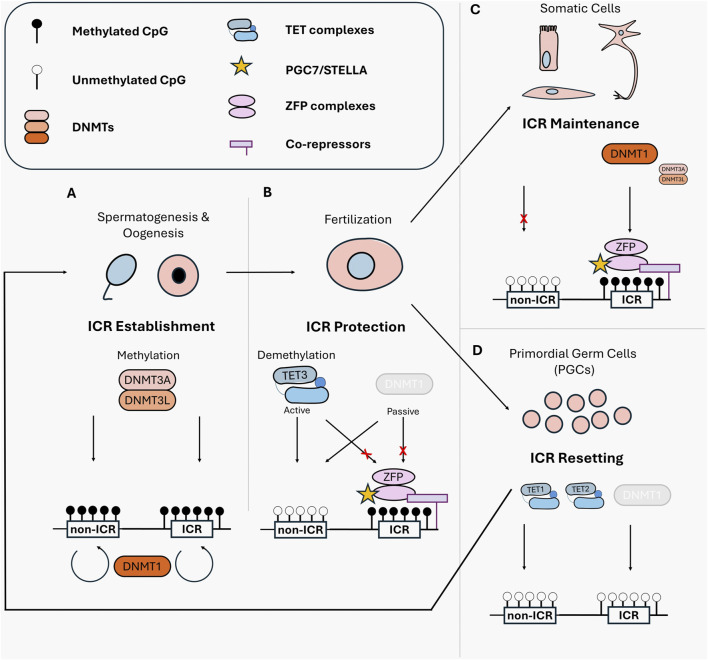
Schematic overview of imprinting dynamics during embryogenesis at ICRs in mammals. **(A)** Establishment during gametogenesis. In oocytes and spermatocytes, the *de novo* DNA methyltransferase DNMT3A (with cofactor DNMT3L) methylate ICRs, creating parent-of-origin imprints. **(B)** Protection of ICRs after fertilization. Following sperm and egg fusion, the zygotic genome undergoes TET3-enzyme mediated active demethylation on the paternal genome and passive demethylation (dilution through replication in absence of maintenance DNMT1 activity) on the maternal genome, while ICRs remain protected by zinc-finger protein (ZFP57/ZNF445) complexes. **(C)** Maintenance of ICR DNA methylation throughout somatic life. In somatic cells, the ICR protecting protein complexes from **(B)** also lead to maintenance of the methylated state through attracting DNA methyltransferase for maintenance, particularly DNMT1. **(D)** Imprinting marks are reset in primordial germ cells (PGCs). Emerging PGCs undergo a second wave of global demethylation by TET1 and TET2 as well as passive demethylation (lack of functional DNMT1 activity) that erases parental imprints, after which they are re-established in a gamete-specific manner (see panel A).

In mammals, these sex-specific DNA methylation patterns are established during gametogenesis, primarily by the *de novo* DNA methyltransferase (DNMT) DNMT3A and its cofactor DNMT3L, and further maintained by DNMT1, ensuring that the imprinting pattern appropriate for the future parent’s germline is set for the next-generation (Figure 1A) [13]. The establishment of these DNA methylation marks is tightly regulated by zinc finger proteins, particularly ZFP57 and ZNF445 [14]. Upon fusion of the gametes during fertilization, the epigenome of the zygote is largely reset during early embryogenesis (pre-implantation), with both passive and TET-enzyme (primarily involving TET3) mediated active demethylation of paternal and maternal genomes, to establish a blank slate for later tissue specific epigenetic profiles [15]. However, ICRs are protected from DNA demethylation in the early embryo by ZFP57 together with the PGC7/STELLA complex and other chromatin-associated factors (Figure 1B). In somatic tissues, these ZFPs continue to recruit DNMTs (particularly DNMT1, but also DNMT3A/3B) for DNA methylation maintenance, ensuring stable imprinted expression patterns throughout life (Figure 1C) [15]. A notable exception are the primordial germ cells (PGCs). Whereas their ICRs are initially protected against DNA methylation, they are subject to a second round of demethylation later in embryogenesis (during early gametogenesis), mediated in part by TET1 and TET2. This time it leads to removal of the parent-of-origin-specific epigenetic marks, to be replaced with sex-specific marks to establish genomic imprinting in the next-generation (Figure 1D) [16].

### Other epigenetic mechanisms (co-)regulate imprinting

**FIGURE 2:**
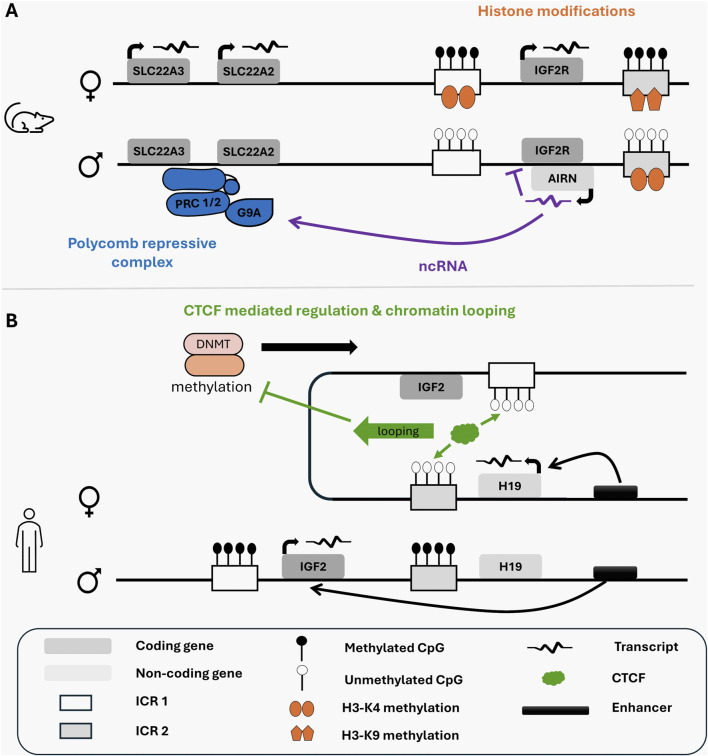
Alternative epigenetic mechanisms establishing or facilitating imprinting, illustrated by regulation of the *IGF2R*/*AIRN* locus in mice and of the *IGF2*/*H19* locus in humans. **(A)** *IGF2R* and *AIRN* regulation in mice. *IGF2R* is expressed from the maternal allele, while the antisense *AIRN* transcript is produced from the paternal allele. Next to DNA methylation, histone modifications coregulate this locus. On the maternal allele, H3K4 methylation at the *IGF2R* promoter (ICR 1) enables gene activation, while DNA methylation and H3K9 methylation at the *AIRN* promoter (ICR 2) suppress *AIRN*. On the paternal allele, H3K4 methylation at the *AIRN* promoter initiates its transcription, which extends over *IGF2R*, leading to paternal *IGF2R* silencing through DNA methylation and H3K9 modifications. Moreover, this locus is coregulated by ncRNA transcription. The *AIRN* transcript overlaps with the *IGF2R* promoter region, inhibiting initiation when transcribed. Moreover, the *AIRN* transcript recruits the G9A mediated PRC1/PRC2 complex to the distal genes SLC22A3 and SLC22A2, thereby silencing the expression of the paternal allele. **(B)** *IGF2* and *H19* regulation in humans. *IGF2* and *H19* exhibit paternal and maternal imprinting, respectively. On the maternal allele, CTCF binds to the unmethylated *H19* ICR (ICR 1) functioning as a DNA methylation insulator, blocking enhancer access to *IGF2* and promoting *H19* transcription. On the paternal allele, *H19* ICR methylation prevents CTCF binding, allowing the enhancer to activate *IGF2* expression. Moreover, at the maternal allele, CTCF also binds the unmethylated *IGF2* ICR (ICR 2) thus facilitating chromatin looping, which helps to maintain unmethylated *H19* and *IGF2* ICRs while ensuring *H19* transcription.

Other epigenetic mechanisms often play an equally crucial role in imprinting control. For example, histone modifications, such as H3K9me3 and H3K27me3, maintain inactive chromatin at the ICR of the silenced allele, whereas H3K4me3 and H3K27ac marks are present on the active allele, resulting in an imbalanced expression pattern (Figure 2A) [17, 18]. Though these histone modifications typically act in combination with DNA methylation, imprinting can also be controlled by histone marks independent of DNA methylation [19]. Alternatively, non-coding RNAs (ncRNAs), such as small piwi-interacting RNAs (piRNAs), microRNAs (miRNAs) and long non-coding RNAs (lncRNAs) contribute to or are even required for correct imprinting establishment or regulation [20, 21]. Moreover, many of these ncRNAs are found imprinted themselves, e.g., *Mir125b-2* [22], *MEG3* (domain) ncRNAs [21] and *AIRN* [23]. These are often directly involved in imprinting regulation *in cis* [16], with, e.g., *AIRN* silencing the IGF2 receptor gene (*IGF2R*) in mice (Figure 2A) [23].

Furthermore, different epigenetic effects are mediated by additional *cis-*regulatory layers, such as insulator binding by the CCCTC-binding factor (CTCF). CTCF binds unmethylated insulator motifs to establish physically separated DNA domains, largely through chromatin looping. This not only inhibits the interaction between regulatory elements (e.g., enhancer-promoter pairs) located on different domains/loops but also helps to prevent the spreading of epigenetic marks beyond the boundaries of imprinted domains. Consequently, CTCF binding is essential to coordinate the expression of multiple genes in larger imprinted regions, with the well-characterized *IGF2/H19* locus on chromosome 11p15.5 as classic example (Figure 2B) [24–26].

### Additional layers of complexity of imprinting regulation

Genomic imprinting is often further regulated by tissue or cell-type specific epigenetic profiles, with particularly neuronal tissues featuring deviating imprinting patterns [27–29]. A key example is *GNAS*, which exhibits imprinting in the brain, pituitary gland and thyroid but biallelic expression in most other tissues [30]. Moreover, several imprinted genes, such as *MEST* and *IGF2*, exhibit transcript-specific imprinting, particularly due to epigenetically regulated alternative promoter usage, with some isoforms featuring monoallelic expression while others being transcribed from both parental alleles [31]. This may be associated with the observation that many imprinted genes feature multiple imprinting-specific methylated regions [32]. Hence, it remains unclear to which extent the perceived absence of or incomplete imprinting in specific tissues or cell types can be attributed to the expression of alternative transcripts.

Additionally, imprinting may also exhibit species specific patterns. For instance, *IGF2R* is imprinted in mice but typically exhibits biallelic expression in humans, while *OSBPL1A* shows isoform-specific imprinting in bovines but not in other mammals [33]. Similarly, *DLX5* is maternally expressed in humans but escapes imprinting in mice [34].

## Imprinting dysregulation in disease

Dysregulation at imprinted loci can lead to the activation of an allele that should be silent or the silencing of an allele that should be active [4], collectively called “loss of imprinting” or LOI. This dysregulation can be caused by multiple processes, with DNA methylation defects, uniparental disomy (UPD), point mutations and chromosomal aberrations as key mechanisms [35] and is relevant in multiple diseases (Table 1).

**TABLE 1:** Examples of causes and consequences of imprinting dysregulation in humans.

Disease category	Example	Dysregulated imprinted gene(s) (selection)	LOI mechanisms	LOI-related clinical features [references]
Congenital disorders^$^	Beckwith–Wiedemann syndrome	*IGF2, H19, CDKN1C*	Hypermethylation at *H19*‐ICR, paternal UPD 11p15.5, *CDKN1C* loss‐of‐function, mutations	Overgrowth, macroglossia, organomegaly, higher cancer risk [36, 37]
	Prader–Willi syndrome	*SNRPN, SNORD116* cluster	Paternal microdeletion or maternal UPD at 15q11–13	Hypotonia, hyperphagia, hypogonadism, behavioural problems, underdeveloped sex organs [38, 39]
	Angelman syndrome	*UBE3A*	Maternal deletion or paternal UPD of 15q11–13	Developmental delay, seizures, ataxia, intellectual disability [38, 40]
	Silver–Russell syndrome	*IGF2, H19, CDKN1C, MEST, KCNQ1, GRB10*	Hypomethylation at 11p15 *H19*/*IGF2*, multi-locus imprinting disturbance, maternal UPD	Growth failure, severe feeding difficulties, gastrointestinal problems, hypoglycaemia, body asymmetry, motor and speech delay [41, 42]
	Temple syndrome	*MEG3, DLK1*	UPD, epimutations, microdeletions	Pre- and postnatal growth failure, insulin resistance, marked hypotonia and feeding disabilities [43]
	Kagami-Ogata syndrome	*MEG3, DLK1*	UPD, epimutations, deletions	Facial dysmorphism, skeletal abnormalities, growth retardation, and developmental delay [44]
Cardio-vascular and metabolic	Atherosclerosis	*IGF2, H19*	Hypomethylation of *H19*/*IGF2* ICR, biallelic expression	*Smooth-muscle proliferation and plaque progression [45]
	Preeclampsia	*DLX5*	Hypomethylation of *DLX5*	*Earlier-onset, severe maternal hypertension [34]
	Transient neonatal diabetes type 1	*PLAGL1, HYMAI*	Hypomethylation at the maternal *PLAGL1/HYMAI* DMR (6q24), UPD	*Growth restriction, (neonatal) hyperglycaemia [41]
Cancer^$^	Colorectal carcinoma	*IGF2, H19, CDKN1C*	Hypomethylation of *IGF2*, *CDKN1C* promoter hypermethylation	Tumour aggressiveness, metastatic potential, and poor survival [7, 46, 47]
	Hepatocellular carcinoma	*MEST, IGF2, H19*	Genome wide and locus-specific promoter methylation alterations	Rapid tumour growth, vascular invasion, poor survival (*IGF2/H19*) [48, 49]
	Non–small cell lung cancer	*GNAS, HM13, H19, IGF2*	Promoter hypomethylation	Proliferation, metastasis, radiotherapy resistance, poor prognosis [49, 50]
	Wilms tumour	*IGF2, H19*	*IGF2* hypomethylation	Increase tumour size [51]
	Breast carcinoma	*IGF2, DIRAS3, HM13*	*IGF2* and *HM13* hypomethylation	Enhanced proliferation, poor prognosis [7, 52, 53]
	Epithelial ovarian cancer	*PEG3, MEST, IGF2, H19*	Hypomethylation, *H19* derived oncomiR upregulation	Advanced stage, chemoresistance, poor outcome [24, 54, 55]
	Gliomas	*DLK1, PEG3*	Promoter hypermethylation	Reduced p53-mediated apoptosis, higher tumour grade [7, 56, 57]

* Clinical features associated with disease yet not necessarily caused by LOI, itself.

^$^ Congenital disorders associated with higher cancer risk are listed under the former.

### LOI in congenital, neuropsychiatric and metabolic/cardiovascular disorders

A critical window for imprinting dysregulation occurs during embryogenesis. Global DNA demethylation must be balanced by robust maintenance mechanisms to safeguard correct methylation at ICRs [58], and epigenetic imprinting marks should be correctly established during germline development (Figure 1) [6]. During these critical windows, epimutations are, if not triggered, often exacerbated by environmental influences, such as deficiency in folate, an essential methyl donor to establish and maintain DNA methylation [59].

In parallel, genetic aberrations such as point mutations (including microdeletions), copy number alterations (CNA) or UPD occurring prior to or during early development, can disrupt the precise dosage of individual or multiple imprinted genes [60, 61]. The latter may also occur due to mutations affecting key regulators, such as ZFP57 or UHRF1, potentially leading to multi-locus imprinting disturbances, further underscoring the interplay between genetic and epigenetic factors [32, 62]. Early life LOI events often result in congenital developmental disorders, which frequently feature elevated (childhood) cancer risk [35]. Beckwith-Wiedeman Syndrome (BWS) for example, is characterized by excessive foetal growth and an increased risk of childhood cancers due to dysregulated imprinting at the *IGF2/H19* locus [36]. Prader-Willi Syndrome (PWS) and Angelman Syndrome (AS) both result from deletions (∼70%) or UPD (∼25%) affecting the imprinted region 15q11-q13, yet lead to distinct clinical outcomes depending on the parental origin of the affected allele [38].

Beyond congenital disorders, genomic imprinting defects have also been linked to a range of complex diseases, including cardiovascular conditions, neuropsychiatric disorders and cancer. Such defects may arise later in life but may also be set during early development (often leading to a mosaic LOI pattern) and impact disease risk during the life course. For example, altered imprinting of *IGF2* and *DLK1* has been associated with an increased risk of hypertension and coronary artery disease [63]. Dysregulated imprinting was also observed in atherosclerotic plaques, where the imprinted *DLK1-MEG3* domain was extensively hypomethylated, and featured overexpression of multiple clustered miRNAs [45]. Altered methylation of this cluster has been linked to increased susceptibility to type 1 diabetes [64], as well as neurobehavioral traits. In mouse models, for example, imprinting dysregulation at *DLK1*-*MEG3* was found to lead to heightened anxiety-related behaviours in adulthood [65]. Similarly, dysregulation of *GNAS* imprinting has been demonstrated in cognitive disorders, further supporting its neurodevelopmental significance [30]. Moreover, imprinting disruption has been proposed to underly autism spectrum disorder and schizophrenia, as parent-of-origin effects were found to affect cognitive function and behavioural phenotypes [41, 66].

### LOI in cancer

However, among imprinting-associated diseases, cancer represents a frequent outcome of imprinting dysregulation. This dysregulation is typically somatic, i.e., cancer-specific, but may also have been congenital–as is the case for BWS [36] – or have arisen during early development, which leads to mosaic, i.e., tissue dependent, LOI patterns. LOI in cancer is frequently attributed to epigenetic alterations, though this interpretation may sometimes (but certainly not always [52, 67]) be confounded by the presence of CNAs [68]. Similarly, also genome-wide cancer DNA hyper- or hypomethylation may lead to LOI. Hence LOI can also be a passenger phenomenon. Nevertheless, given that many imprinted genes regulate cell growth, differentiation, and apoptosis, their dysregulation can also directly contribute to tumorigenesis.

In general, LOI at the *IGF2/H19* locus is the most extensively documented imprinting alteration in cancer. Here, re-expression of the normally maternally silenced *IGF2* allele and silencing of the maternally expressed *H19* non-coding RNA are assumed to be growth promoting. For example, LOI at the *IGF2/H19* locus is considered a risk biomarker for colorectal cancer: LOI has been documented in nearly 30 percent of patients’ normal colonic mucosa, and LOI detected in blood greatly increases the odds for both colorectal adenoma and carcinoma [46]. *IGF2/H19* LOI has been described in multiple other cancer types, including breast, liver, lung, and Wilms tumour [24, 48, 51, 69], where it was found to be growth-promoting and lead to pro-oncogenic signalling. Moreover, it recapitulates foetal-program proliferation patterns [70], linking LOI in cancer back to the parental conflict hypothesis of imprinting. *IGF2/H19* LOI may be equally relevant from a treatment perspective, as it leads to poorer response to treatment and adverse clinical outcomes [71], making it also a putative predictive and prognostic biomarker.

Next to *IGF2/H19*, multiple other imprinted loci undergo LOI or aberrant methylation in cancer. *DLK1* often features LOI in gliomas and neuroblastomas, promoting tumour progression [56]. Also in glioma, hypermethylation mediated *PEG3* silencing is correlated with a higher tumour grade and beta-catenin accumulation [7, 57], making it a potential prognostic biomarker. In several cancer types, abnormal imprinting patterns have been observed for tumour suppressor *CDKN1C* [6, 37]. *HM13*, an imprinted gene involved in proteolysis of signal peptides, has an equally emerging role in oncogenesis, as it exhibits LOI in multiple cancers, including breast, kidney and lung tumours, and was found to have independent prognostic marker potential [50, 52, 72]. *MEST* is another gene frequently subject to LOI, e.g., in breast, ovarian, and lung carcinomas, with putative prognostic biomarker value [48, 53, 54]. Hence, the precise characterization of imprinting defects is recognized as a valuable strategy for clinical cancer research [3, 15]. Nevertheless, as we will discuss in the next sections of this manuscript, progress in clinical research and translation is severely hampered by methodological limitations.

## Established methods to study imprinting and its dysregulation

In many model organisms, such as the mouse, imprinting can be studied in a cost-efficient manner through the characterization of the largely heterozygous progeny created by mating parents from different inbred lines. By genotyping the parents and profiling the genotypes and RNA-seq profiles of a few progeny samples, it can be evaluated whether allele-specific expression effects in the offspring can be attributed to the parent of origin. The presence of imprinting can be validated by applying reciprocal crosses of the parent lines, as this should lead to the other allele being expressed in the offspring [73]. Subsequently, for normally imprinted loci, a similar strategy can be used to evaluate the presence of imprinting deregulation in murine models of human disease. Alternatively, one can evaluate the impact of knock-out or overexpression of imprinted genes on disease risk.

Nevertheless, as outlined in a previous section, genomic imprinting and its regulation is often species-specific, making human-specific experiments necessary for clinical studies. For example, the retinoblastoma gene *RB1* is imprinted in humans but not in mice [74], and attempts to generate an imprinted mouse model for this gene were unsuccessful [75]. Hence, existing murine models of retinoblastoma lack this additional level of complication. Similarly, *IGF2* LOI is common and an epigenetic driver in Wilms tumour. Children heterozygous for germline mutations in the Wilms tumour gene (*WT1*) are predisposed to the development of Wilms tumour. Heterozygous mice, however (homozygous mutations are lethal), do not feature tumour development, unless *Igf2* is overexpressed. In humans, *IGF2* overexpression and *WT1* mutations frequently co-occur, which has been attributed to the synteny of *WT1* and *IGF2* on the short arm of chromosome 11. Paternal isodisomy of 11p, resulting in two copies of the paternally expressed IGF2 allele, is often observed in *WT1* mutant tumors, and both aberrations may originate from a single, complex genetic event. In mice, however, *Wt1* and *Igf2* are not syntenic, meaning these two alterations require two independent events, making murine models less representative due to their different genomic architecture [76].

Therefore, in the next subsections, we elaborate on relevant gene expression and epigenetics strategies to study imprinting and LOI specifically in humans. This will include genome-wide approaches for clinical research, but also locus-specific/targeted tests as basis of clinical biomarker assays. A summary of these methods is provided in Table 2.

**TABLE 2:** Summary of epigenetic and transcriptomic methods for (loss of) imprinting assessment.

Strategy	Methods	Characteristics relevant to imprinting research
Sequencing-based transcriptomic analyses	Bulk RNA-seq [52, 77]	Imprinting studies in parent-offspring trios and/or case-control designs
	Targeted RNA-seq (PCR- or hybridization-based) [78]	Expression based imprinting analysis at regions of interest
	scRNA-seq (10x Genomics, SMART-seq) [53, 79]	Single-cell resolution profiling of expression and (epi)genomics
	Long-read RNA-seq (nanopore, PacBio) [80]	Haplotype-resolved epigenetic and (full-length) transcriptomic analyses
Epigenetic analyses	WGBS [81]	Genome-wide, base-resolution DNA methylation profiling
	EM-seq [82]	Enzymatic alternative to bisulfite conversion with reduced DNA degradation
	RRBS [83, 84]	Single-base resolution DNA methylation profiling at CpG-rich regions
	BSP [85]	Targeted, base-resolution methylation analysis (sequencing-based)
	MSP, MethyLight [86]	Targeted, without base-resolution
	MeDIP-seq, MethylCap-seq, MBD-seq [86, 87]	Enrichment of methylated DNA followed by sequencing
	DNA-methylation arrays (Infinium HumanMethylation, MethylationEPIC) [88]	Cost-effective, genome-scale (targeted) DNA methylation profiling; no allelic read-out, (+/−) base-resolution
	COBRA [89]	Targeted, without base-resolution
	MS-MLPA [90]	Combines copy number alteration and DNA methylation results, straightforward multiplexing for imprinting syndromes
Spatial methods	QCIGISH [12, 91]	Chromogenic *in situ* hybridization quantifying allele-specific transcription

Abbreviations: scRNA-seq, single-cell RNA sequencing; SMART-seq, Switching Mechanism At the 5′ end of RNA Template single-cell total RNA sequencing; WGBS, Whole-Genome Bisulfite Sequencing; EM-seq, Enzymatic Methylation sequencing; RRBS, Reduced Representation Bisulfite Sequencing; BSP, Bisulfite Sequencing PCR; MSP, Methylation Specific PCR; MeDIP-seq, Methylated DNA Immuno Precipitation sequencing; MBD-seq, Methyl-Binding Domain sequencing; COBRA, Combined Bisulfite Restriction Analysis; MS-MLPA, Methylation-specific Multiplex Ligation-dependent Probe Amplification; QCIGISH, Quantitative Chromogenic Imprinted Gene *In situ* Hybridization.

### Expression-based strategies

**FIGURE 3:**
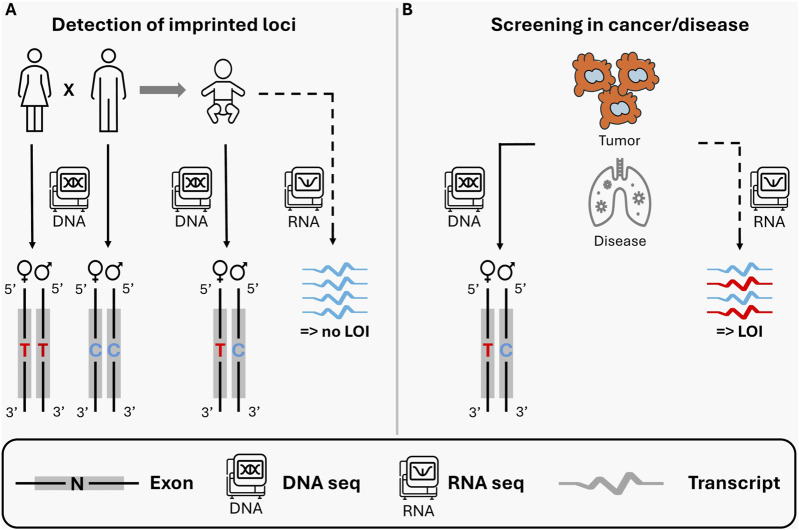
Parent–offspring trio based (loss of) imprinting detection. **(A)** Detection of imprinted loci. By genotyping both parents and offspring, heterozygous sites can be identified. Given the identification of heterozygous loci, imprinted loci can be detected by the absence of either paternal or maternal allele expression in RNA sequencing data for the offspring. Imprinting is present when the expressed allele is consistently maternal or paternal across trios. **(B)** Screening of imprinting in cancer and disease. Using known imprinted loci (as inferred in A), we can detect loss of imprinting (LOI) in RNA sequencing data of the cancer/diseased sample. Note that genotyping of the affected individual is required, as LOI calls for a locus under study can only be made for heterozygous individuals.

The most straightforward strategy to characterize imprinting in a human context relies on combining genomics and transcriptomics data in a parent-offspring trio setting [77, 78]. More specifically, by genotyping the offspring, heterozygous loci can be identified, upon which RNA-seq of the tissue under study can be used to evaluate whether only a single allele is expressed for these loci, compatible with imprinting [73]. By comparison with parental genomics data, it can subsequently be evaluated from which specific parent the expressed allele is inherited. Loci consistently featuring monoallelic expression of the paternal or maternal allele across trio’s are then considered to feature genomic imprinting (Figure 3A). Upon characterization of imprinting in a healthy tissue, the same loci in the corresponding diseased tissues can be evaluated to identify imprinting defects, without the need for parental data of the cases (Figure 3B) [50, 53].

Though considered the gold standard for research, this strategy is limited by the need for heterozygosity to enable the discrimination of both alleles in the offspring [73, 77]. This implies that sufficiently large numbers of trios should be considered to ensure that multiple heterozygous offspring can be identified for reliable imprinting. Combined with the need for both genomics and transcriptomics data, this leads to expensive designs [78, 81]. In part, this can be mitigated by relying on less comprehensive assays for genotyping, such as genotyping arrays, yet this automatically results in an incomplete overview. Similarly, total RNA-seq is necessary to also capture non-polyadenylated transcripts, but most studies only focus on poly-A enrichment-based RNA-seq. When only interested in few loci, targeted RNA-sequencing upon PCR- or hybridization-based enrichment can improve the sensitivity and specificity of imprinting (dysregulation) assessment. Such a targeted assay can in principle also be implemented in a clinical setting yet would only be useful for subjects featuring heterozygous SNPs in the targeted region.

The immediate consequence of the high cost for comprehensive imprinting screening is that studies on imprinting dysregulation often rely on compendia of known imprinted loci, such as Geneimprint (http://www.geneimprint.com). Yet, such compendia are most likely incomplete due to the challenges outlined in the previous paragraph. Moreover, they are often not completely valid for the case-study at hand due to (species and) tissue-specific imprinting effects. As an alternative strategy, case-control studies often first screen for “candidate” imprinted loci in controls, followed by further analysis of solely those loci in cases. This strategy abolishes the requirement of trio data and is applicable on the large number of datasets with matching RNA-seq and genotyping data in public repositories. Yet, it often leads to loci falsely detected as imprinting [73], particularly due to allele-specific expression effects independent of parental origin, e.g., random monoallelic expression (RME) of human leukocyte antigen genes.

For sufficiently large designs, DNA-based genotyping is not even strictly necessary for imprinting screening: population genetics dictates how many heterozygous individuals are expected for a given locus, making imprinting a likely option when virtually none are found in the RNA-seq data. Upon application of this principle in breast tissue, far more loci were detected as putatively imprinted than when also genotyping data was taking into account, as many loci were not (sufficiently) covered by the latter [52].

### Epigenetics-based strategies

As genomic imprinting is tightly regulated by epigenetics, many studies solely focus on epigenetic data rather than expression data, DNA methylation in particular [15]. Most assays, such as whole-genome bisulfite sequencing (WGBS) [81], rely on next-generation sequencing (NGS) of DNA upon the conversion of non-methylated cytosines to uracil, providing a direct means to evaluate whether the degree of DNA methylation approximates/deviates from the anticipated 50% for imprinting. These methods also capture SNPs, enabling one to differentiate between both alleles, though complicated by the fact that the conversion of unmethylated cytosines interferes with SNP detection. Additionally, the requirement for sufficiently large high-quality and high-purity tumour samples, as well as bisulfite treatment induced DNA degradation, poses significant challenges for bisulfite sequencing in cancer research [92].

This has led to the development of low-input bisulfite sequencing protocols suitable for tumour biopsies [93] and enzymatic rather than bisulfite-based conversion (EM-seq) [82, 94]. Moreover, bait-based capture of genomic regions of interest prior to sequencing [8, 95] and reduced representation bisulfite sequencing (RRBS) [83] strategies have been developed to avoid the high cost of whole-genome bisulfite sequencing. In a clinical setting, the principle of cytosine conversion can be used to assess LOI associated methylation changes at selected loci, even though the (at most) limited allele-specific read-out entails rigorous assay design to avoid tumour purity related bias. Relevant methods include by bisulfite sequencing PCR (BSP) [96], (quantitative) methylation-specific PCR ((q)MSP) [97], MethyLight [98] and Combined Bisulfite Restriction Analysis (COBRA) [89]. Alternative methods rely on methylation-sensitive restriction enzymes, such as Methylation-specific (digital) Multiplex Ligation-dependent Probe Amplification (MS-MLPA) [90]. MS-MLPA also assesses copy number changes (the original MLPA) and can be easily multiplexed, making it the most widely used methodology for the diagnosis of imprinting syndromes [99].

Rather than relying on unmethylated cytosine conversion and sequencing (a substantial part of) the full genome, one can also opt to solely sequence methylated DNA through enrichment strategies, based on antibodies like methylated DNA immunoprecipitation (MeDIP-seq) [100] or methyl-binding domains (MBD) (MethylCap-seq/MBD-seq) [101]. Although these methods have been successfully used to study imprinting they lack single-CpG resolution and also capture unmethylated background [102]. Therefore, they have sometimes been complemented with other techniques of post enrichment to increase resolution for imprinting research [87].

A very cost-efficient alternative is the use of DNA methylation arrays, particularly the Illumina Infinium HumanMethylation assay series (e.g., HumanMethylation450K and the more recent MethylationEPIC platform), which target many known imprinted loci and have hence frequently been used in imprinting research, e.g., [88]. Also, custom Infinium arrays targeting the human “imprintome” have been developed and reportedly achieved high diagnostic accuracy in detecting aberrant *IGF2/H19* methylation in thyroid cancer [103]. The main caveat is that these arrays do not measure the methylation status in an allele-specific manner, making it unclear whether DNA methylation alterations reflect dysregulated imprinting or altered cell-type composition. Nevertheless, available ICR coordinates have been largely mapped on the Infinium assay, making this strategy a very straightforward option for at least preliminary evaluation [104].

In conclusion, each strategy mentioned has its own advantages and drawbacks. Yet, all of them share several disadvantages that further limit their practical use, both regarding the interpretation of results and translation to the clinic. In the next section, we further elaborate on these disadvantages and how they can be addressed by novel technologies.

## Methodological challenges and novel technologies for cancer imprinting research

### Outstanding challenges in cancer imprinting research

In its canonical form, LOI can either refer to re-expression of the originally silenced allele or silencing of the originally expressed allele. In cancer studies, it is anticipated that, upon LOI, growth promoting imprinted genes (with typically expression of the paternal allele) will feature biallelic and hence overexpression, whereas growth limiting genes (with typically expression of the maternal allele) become biallelically and thus completely silenced. In terms of DNA methylation-based strategies, this translates into hypo- or hypermethylation of ICRs of growth promoting respectively limiting imprinted genes. Yet, often, observations in cancer do not fit within this paradigm. For example, cancer-specific biallelic expression of a normally imprinted gene was often found to be accompanied with lack of upregulation, or even downregulation of the imprinted gene. Moreover, in some cases, cancer-specific biallelic expression was found for putative growth-suppressing genes, *i.e.*, where normally solely the maternal allele is expressed, e.g., [52]. Most likely, such observations can be explained by technical and biological complications hindering the evaluation and interpretation of the imprinting status in a cancer context, implying a major bottleneck for translation to a clinical setting.

**FIGURE 4:**
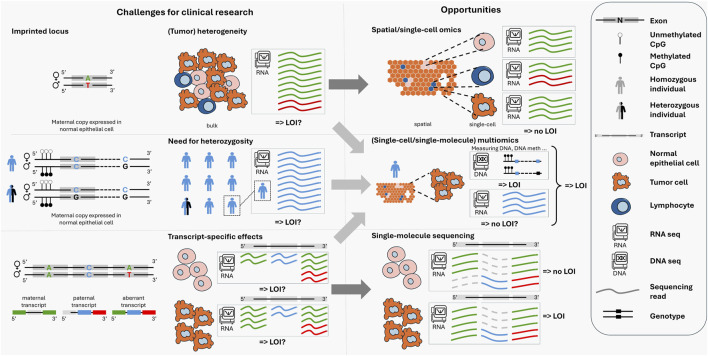
Schematic overview of challenges for clinical (loss of) imprinting (LOI) research and the opportunities due to advantages in state-of-the-art sequencing techniques. We discern three major challenges: i) (tumour) heterogeneity, ii) the need for heterozygosity, and iii) transcript-specific effects, which can be largely addressed (integrating) spatial/single-cell and single-molecule sequencing strategies.

A first challenge is that most studies rely on bulk tumour samples, which may include substantial fractions of non-tumour cells, such as tumour associated fibroblasts or infiltrating immune cells, reaching, e.g., up to 50% in renal cancer [105]. If one of those admixing cell types features different imprinting patterns than those of the original tissue and derived tumour, this may lead to incorrect LOI calls. For example, non-imprinted infiltrating immune cells within a tumour may appear as cancer-specific LOI, just as biallelic expression in normal fibroblasts may provide a background misinterpreted as LOI when the imprinted gene is simply downregulated in cancer (Figure 4). The latter may, e.g*.*, explain why biallelic expression has often been observed for genes featuring expression downregulation [52]. Note that similar issues arise when studying DNA methylation. Hence, tumour purity has frequently been put forward as an explanation for hard-to-interpret tumour specific observations, e.g., [52, 53].

A second challenge is that it should be possible to discriminate paternal from maternal alleles to evaluate (loss of) imprinting, implying a need for heterozygosity (Figure 4). Therefore, most screening strategies combine genotyping to identify heterozygous individuals for the locus under study with allele-specific quantification in the latter [106, 107]. This implies that the power to detect (loss of) imprinting is not solely determined by sample size, but also varies across the genome as a function of genotype frequency [107]. Though relevant for scientific studies, this issue is a major bottleneck for clinical LOI assays. Indeed, if one would assess allelic expression at a given SNP locus, LOI cannot be inferred for homozygous individuals. Prior enrichment for and RNA-sequencing of a full-length gene increases the chance of - but does not guarantee - identifying heterozygous SNPs, and requires knowledge of any transcript-specific imprinting.

For clinical assays quantifying the degree of DNA methylation, SNPs are not strictly required to call LOI events as they typically entail a clear shift from 50% to about 0 or 100% methylation. Yet, in practice, tumour impurity and other sources of bias impede accurate quantification, making SNPs key to evaluate whether a methylation shift is indeed allele-specific (Figure 4). This leads to similar problems as for expression-based assays, possibly aggravated by artificial SNPs introduced by bisulfite (or enzymatic) conversion.

In addition to these technical limitations, also biological phenomena complicate the interpretation of imprinting analyses. Transcript-specific imprinting has been observed for multiple loci, e.g., in *MEST* and *IGF2* [31, 108]. Upon imprinting dysregulation, this may lead to the generation of alternative transcripts rather than straightforward allelic reactivation/silencing, and hence inconsistent LOI across the gene’s SNPs (Figure 4). Moreover, NGS based gene expression statistics may fail to discriminate differential expression from a shift to different transcripts, respectively [53].

The widespread presence of CNAs in cancer is another factor impacting LOI interpretation [82]. Next to causing false-positive LOI calls, e.g., when solely based on deviation from 50% methylation, common CNAs can also lead to LOI at imprinted loci, e.g., due to a high rate of deletions, or through the gain of an originally silenced allele without its correct epigenetic context [68]. Nevertheless, attributing clinical relevance to these LOI events is not straightforward given the many neighbouring genes also recurrently targeted by the common CNA.

### Single-cell omics strategies to resolve LOI in cancer

Single-cell RNA-sequencing (scRNA-seq) approaches have revolutionized our ability to resolve tumour heterogeneity by profiling individual tumour cells without the interference of other potentially differently imprinted cell types. Nevertheless, many platforms such as 10x Genomics [109] by default focus on sequencing 5′ or 3′ transcript ends, thereby missing a large number of the SNPs required to separate alleles. Other technologies, such as SMART-seq [79], enable more comprehensive LOI studies by characterizing the full-length transcriptome. For subsequent imprinting data-analysis, one can use specialized data-analytical pipelines such as BrewerIX and DAESC [110, 111]. Complementing single-cell transcriptomic methods, single-cell bisulfite sequencing (scBS-seq) enables DNA methylation profiling at the single-cell level with allele resolution, thereby dissecting parent-of-origin methylation patterns at key imprinted regions [85, 112].

Yet, it should be noted that the (epi)genomic single-cell assays are technically hampered by the presence of two gene copies per cell (CNA withstanding), further aggravating single cell data sparsity. This leads to a poor genome-wide character, with, e.g., at most about 15% of all CpGs covered in scBS-seq, making it virtually impossible to perform cell-level LOI analysis [113]. A similar resolution problem arises for scRNA-seq, where irregular intermittent expression of each allele (transcriptional bursting) cannot be discriminated from ASE effects at the single cell level [114]. This resolution problem can be mitigated through a pseudobulk strategy, where the data from solely tumour cells (or subpopulations) is combined for analysis and further processed as (pure tumour) bulk data [110, 111]. Despite the major advantage of single-cell LOI studies by addressing tumour purity, other listed limitations remain. Moreover, cost remains a major bottleneck, particularly for population-level cancer studies where LOI may be only present in a subset of cancers evaluated. Coupling default large-scale bulk-strategies for candidate LOI screening with single-cell strategies of a limited number of tumour samples for better evaluation and interpretation has been proposed as a cost-efficient alternative [53, 115].

### Targeted spatial and single-molecule omics strategies to resolve LOI in cancer

Similar to single-cell approaches, spatial transcriptomics and related protocols have the potential to study imprinting (dysregulation) within a tissue in a genome-wide manner, yet with currently even more technical limitations. However, targeted spatial methods have the potential of high-resolution allele-specific visualization, which can generate a straightforward LOI readout. A key example is Quantitative Chromogenic Imprinted Gene *In Situ* Hybridization (QCIGISH), an RNA-FISH based technique that targets intronic RNA to visualize active transcription sites [91, 116]. Since expression status can be individually visualized for both gene copies (or more, in case of CNA), QCIGISH enables the detection of LOI when applied on imprinted genes, even in a homozygous background. This strategy offers major clinical potential, as it addresses aforementioned problems with tumour purity, CNAs, and heterozygosity. Indeed, the QCIGISH technology is the cornerstone of Lisen Imprinting Diagnostic’s tests for early cancer detection and monitoring (www.lisenid.com), the sole dedicated clinical cancer imprinting tests currently on the market. This approach was validated in cancer patient cohorts, where QCIGISH achieved near-perfect sensitivity and specificity for early-stage lung cancer detection, but also could be used for cervical cancer risk stratification, confirming the clinical utility of imprinting-based biomarkers [50, 117]. Nevertheless, as for genome-wide single-cell and spatial methods, QCIGISH lacks the isoform resolution required to distinguish between closely related transcripts.

Currently, solely long-read sequencing (LR-seq) technologies offer the possibility to accurately resolve transcript-level imprinting patterns in cancer, e.g., due to aberrant alternative splicing or promoter switching. However, the complete characterization and quantification of all common and rare full-length transcripts per cell type remains a major challenge, even in normal cells [80]. Most LR-seq imprinting studies rather focus on DNA methylation, since both Oxford Nanopore and PacBio provide direct DNA methylation read out during genome sequencing, thereby avoiding bisulfite and enzymatic conversion related problems (DNA fragmentation, alignment issues). Moreover, the generated genomics data also allows to directly infer CNAs or UPD, and practically ensures the presence of SNPs to discriminate between alleles. Consequently, this strategy has been successfully used to identify the pathogenic mechanism underlying congenital imprinting syndromes, typically through more cost-efficient targeted LR-seq [39]. Even though LR-seq applications in a cancer imprinting context remain limited and potentially confounded by the typical bulk approach, it remains a powerful strategy for LOI screening and to expand our understanding of transcript-specific imprinting mechanisms [80].

## Discussion

Continuous improvements in single-cell, spatial and long-read platforms are expanding the scope of imprinting research, transforming our ability to screen the epigenome and transcriptome at ever increasing resolution. Nevertheless, another leap is expected when these strategies are successfully integrated, as already performed in other contexts [118]. Indeed, applied on a cancer imprinting context, this will largely resolve remaining issues with non-cancer cells in bulk tissues (through the single-cell/spatial component) and the need for heterozygosity/transcript-level resolution (through the long-read component). Ideally, single-cell/spatial long-read transcriptomics and epigenomics can even be assessed at the same time through a multi-omics approach (Figure 4). Together, this will not only greatly enhance our ability to accurately characterize LOI and its clinical relevance, but also to identify the underlying mechanisms.

In a next step, the obtained insights can be translated into optimized clinical tests that circumvent imprinted gene-specific limitations regarding heterozygosity, tumour purity, transcript-specific effects etc. currently impeding a correct interpretation. For example, once transcript-specific LOI effects have been characterized through a single-cell single-molecule strategy, QCIGISH probes can be optimized to specifically target LOI-associated transcripts. In other cases, LOI-associated DNA methylation or transcript differences may prove sufficiently tumour-specific to allow for a targeted bulk assay, perhaps even through liquid biopsy. Moreover, LOI loci are candidate targets for future therapeutic intervention. For example, epigenome editing successfully led to the activation of the normally silenced (maternal) *SNRPN* copy, and was proposed as therapy for compensating for the loss of the normally active paternally expressed allele in Prader-Willi syndrome [11]. In a similar vein, activation of the normally silenced paternal *UBE3A* allele was achieved in Angelman syndrome neuronal cells, aiming to compensate for the loss of the normally expressed maternal *UBE3A* allele [9, 10]. Even though extending this type of therapy to a cancer context will face additional challenges, it is clear that the integrated characterization of LOI holds significant promises for translation to the clinic as biomarkers and beyond.

In conclusion, this review demonstrates that a thorough understanding of genomic imprinting and its dysregulation is essential for deciphering the role of LOI in cancer. Currently, particularly bulk NGS epigenomics and transcriptomics strategies are being used, yet tumour impurity, transcript-specific expression and the need for heterozygosity are major bottlenecks for clinical cancer imprinting research. We therefore discussed how recent technological advances in single cell sequencing, spatial transcriptomics and long-read sequencing address these challenges, thereby greatly improving our ability to detect these imprinting defects and to provide valuable insight into the mechanisms of deregulation. In turn, such understanding holds significant clinical potential by guiding the development of clinical cancer markers and even therapies.
